# Landscape of *TET2* Mutations: From Hematological Malignancies to Solid Tumors

**DOI:** 10.1002/cam4.70792

**Published:** 2025-03-21

**Authors:** Zoë L. Hawking, James M. Allan

**Affiliations:** ^1^ Newcastle University Centre for Cancer, Translational and Clinical Research Institute Newcastle University Newcastle upon Tyne UK

**Keywords:** cancer, methylation, somatic mutation, survival, *TET2*

## Abstract

**Background:**

The ten–eleven translocation (TET) enzyme family is a key regulator of DNA methylation, responsible for the conversion of 5‐methylcytosine to 5‐hydroxymethylcytosine to promote locus‐specific demethylation. Thus, it is not surprising that loss or attenuation of TET enzymes is implicated in genomic hypermethylation and transcriptional reprogramming that drives cancer development. Somatic mutations in *TET2* are observed in the bone marrow of 5%–10% of healthy adults over 65 years of age, imparting a hematopoietic stem cell advantage and subsequent clonal hematopoiesis of indeterminate potential (CHIP), a condition which is associated with increased risk of myeloid malignancy. Somatic *TET2* mutations are frequently reported in myeloid disorders, including myelodysplastic syndrome (MDS) and acute myeloid leukemia (AML). Evidence suggests that *TET2* mutations also affect prognosis in myeloid leukemia and other hematopoietic malignancies. However, there is a paucity of collated data on the frequency of *TET2* mutations in solid human cancers.

**Objectives:**

We review the published literature on *TET2* mutation in human solid cancers and explore their frequency and impact on patient outcomes.

**Results & Conclusions:**

Somatic *TET2* mutations are reported in numerous solid human cancers, including those arising in the skin, lung and prostate. Many of the somatic *TET2* mutations reported in solid cancers are recurrent, suggesting functionality. There is also evidence to suggest that somatic *TET2* mutations affect prognosis in solid human cancers.

## Graphical abstract

Somatic TET2 mutations are reported in numerous solid human cancers, including those arising in the skin, lung, and prostate, although there is a paucity of data on their frequency in these settings compared to hematological malignancies. TET2 mutations affect prognosis in myeloid leukemia and other hematopoietic malignancies, but little is known about their impact on solid tumors. Here, we review the data on TET2 mutation in human solid cancers and explore their impact on prognosis and patient outcome.

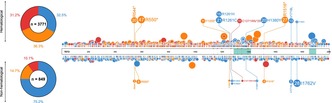

## 1 Introduction

**FIGURE 1:**
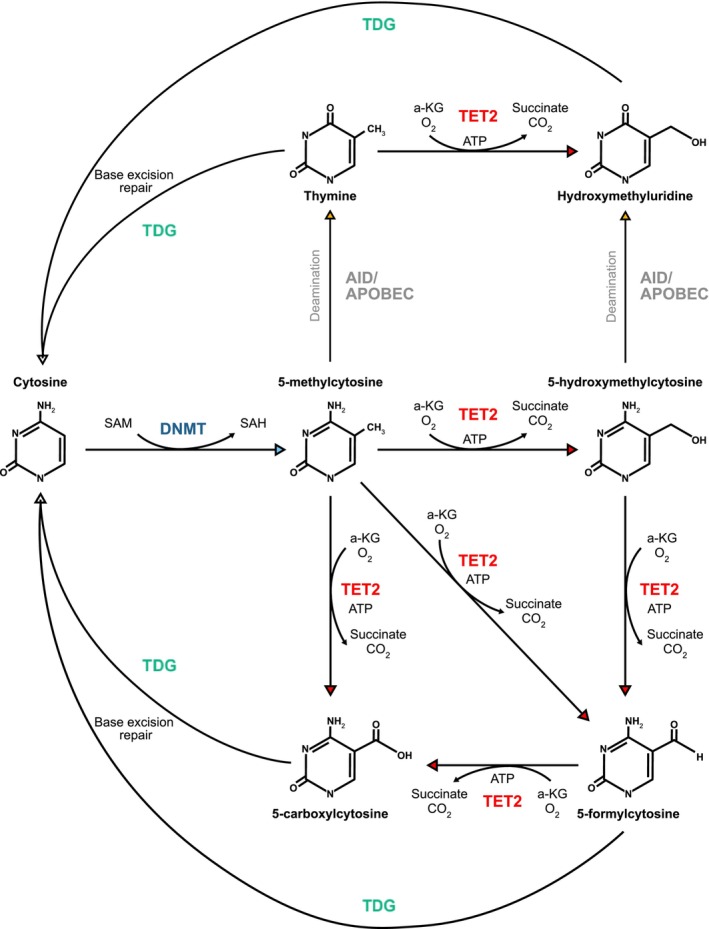
DNMT‐ and TET‐mediated DNA methylation and demethylation. DNA methylation is established and maintained by DNA methyltransferases (DNMTs) through covalent addition of a methyl group to the carbon‐5 position of cytosine, generating 5‐methylcytosine (5mC). TET enzymes catalyze 5mC demethylation via oxidation of the methyl group, generating 5hmC. TET proteins can further oxidize 5hmC to form 5‐formylcytosine (5fC) and 5‐carboxylcytosine (5caC). Activation‐induced deaminases (AIDs) or apolipoprotein B mRNA editing enzyme catalytic polypeptides (APOBECs) also demethylate 5mC via deamination. Thymine DNA glycosylase (TDG) then triggers base excision repair (BER) during cell replication and allows regeneration of unmethylated cytosine.

The functional interplay between DNA methyltransferases (DNMTs) and the three TET enzymes (TET1, TET2, and TET3) is largely responsible for the control of DNA methylation and demethylation, respectively. DNMTs, including DNMT1 and DNMT3A, transfer a methyl group from S‐adenosylmethionine (SAM) to the carbon‐5 position of cytosine, generating 5‐methylcytosine (5mC) within the CpG dinucleotide [1]. Genomic methylation frequently results in global transcriptional repression, although there are reports of hypermethylation associating with increases in transcription at specific loci. As such, the acquisition of a hypermethylation phenotype has a locus‐specific effect on transcription; however, there is a general trend toward transcriptional repression in most reported models [2, 3]. Cell division results in passive DNA demethylation where functional DNMT1 is absent at newly synthesized CpG sites as cells undergo replication [4]. Active demethylation was first reported in mouse embryonal development by Mayer et al. in 2000 [5], where it was established that the paternal mouse genome was significantly demethylated prior to the onset of DNA replication [5]. The mechanism of this demethylation pathway was not discovered until 2009, when it was revealed that TET enzymes oxidize 5mC to 5hmC, and high levels of 5hmC accumulate in numerous tissues prior to the restoration of unmethylated cytosine [6]. TET enzymes are responsible for the further oxidation of 5hmC, forming 5‐formylcytosine (5fC), and finally 5‐carboxylcytosine (5caC) [1, 7]. Thymidine DNA glycosylase (TDG) can remove 5fC and 5caC from DNA, triggering base‐excision repair (BER) and the subsequent reintroduction of unmethylated cytosine into the genome [7] (Figure 1). Another process known to actively demethylate DNA involves activation‐induced cytidine deaminase (AID) and apolipoprotein B mRNA editing enzyme catalytic polypeptide 1 (APOBEC1), which deaminates 5mC to thymine via AID/APOBEC‐mediated DNA deamination [4]. The resultant T/G mispair triggers BER to reinstate cytosine to its unmethylated state (Figure 1). However, TET‐mediated demethylation remains the predominant pathway [8].

TET2‐mediated changes in methylomic and transcriptomic profiles are inherently variable across different tissues, and thus the biological consequences of TET2 mutation vary. Although loss of function mutations in TET2 primarily induce a shift towards global genomic hypermethylation [3, 9, 10], TET2 knockout models in AML have also described a notable level of hypomethylation at specific genomic sites [3]. Further studies are required to elucidate the downstream effects of TET2 loss in nonmyeloid cancers and determine the biological impact.

## 2 Landscape of *TET* Mutations in Human Cancer

### 2.1 Distribution of TET Mutations

**FIGURE 2:**
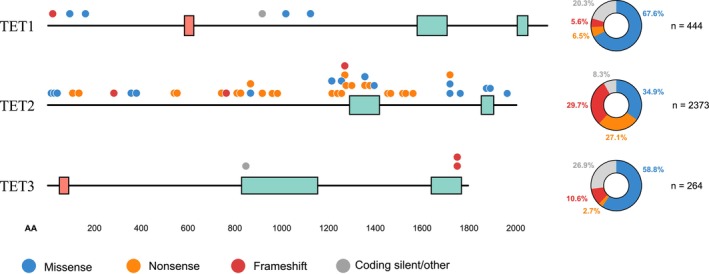
Most frequent TET mutations in human cancer. Recurrent TET mutations displayed in relation to the TET protein structure for TET1, TET2, and TET3. For simplicity, only the catalytic domain (green boxes) and CXXC domain (pink boxes) are highlighted. The top 1% of the most frequently observed mutations based on data from the Catalogue of Somatic Mutations in Cancer (COSMIC) GRCh37 v96 database [8] are represented for each TET, denoted by a circle. Circle color denotes the type of mutation, and pie charts display the total number of mutations seen in each TET protein.

Downregulation of TET protein expression and 5hmC loss are considered epigenetic hallmarks of human cancers [11]. The majority of *TET* mutations affect the *TET2* gene, with 2373 unique *TET2* mutations reported in the Catalogue of Somatic Mutations in Cancer (COSMIC) GRCh37 v96 database [12]. Recurrent *TET2* mutations are distributed across the gene, while recurrent mutations in *TET1* primarily affect the N terminus, and *TET3* mutations cluster at the C terminus. A considerable number of missense and nonsense mutations reported in *TET2* cluster around the C‐terminal catalytic domain (Figure 2), with the potential to affect *TET2* function [11].

**FIGURE 3:**
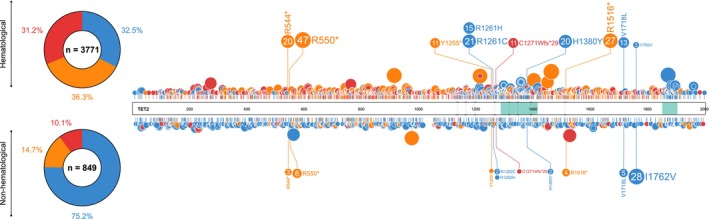
Distribution of missense, nonsense, and frameshift TET2 mutations reported for hematological and nonhematological malignancies. Green areas indicate the location of oxygenase domains on TET2 protein, and dashed lines represent the intersection between exons (exons 3–11). Blue, missense mutation; orange, nonsense mutation; red, frameshift mutation. Noncoding mutations, untranslated region (UTR) mutations, and in‐frame deletions/insertions are not shown. Donut plots highlight the percentages of each mutation type for hematological malignancies (*n* = 3771), including both myeloid and lymphoid, and nonhematological malignancies (*n* = 849). Mutations reported in the Catalogue of somatic mutations in Cancer (COSMIC) GRCh37.COSMIC v96 database [8] in hematological malignancies (top) and nonhematological malignancies (bottom) are shown in lollipop plot. Only mutations repeatedly reported in both hematological and nonhematological settings are highlighted (*n* > 10 in either hematological or nonhematological, with *n* ≥ 1 of the same mutation in the other setting). Circles indicate the amino acid residue location of the mutation, and circle size corresponds to the number of reported mutations at each site. Lollipop plots were created using ProteinPaint web tool developed by St. Jude Children's Research Hospital prior to final figure configuration.

*TET2* mutations are more widely reported and better understood in the context of myeloid disease than in solid tumors. Of the TET2 mutations (only accounting for frameshift, nonsense, and missense mutations) reported in COSMIC across all human cancers, 71% (2074/2923) occur in hematological malignancies, with the remaining 29% (849/2923) reported across a plethora of solid tumors. Over half of the TET2 mutations in hematological malignancies occur in those of a myeloid lineage (65%), with the remaining 35% occurring in lymphoid disease [12]. In hematological malignancies, there is a clear clustering of mutations around the TET2 oxygenase domains, a phenomenon not mirrored in solid tumors (Figure 3). Frameshift, nonsense, and missense *TET2* mutations have a near equal distribution amongst hematological malignancies; however, over 75% of *TET2* mutations reported in solid tumors are missense mutations (Figure 3), and many of these are single‐case reports. As such, it is likely that many of these missense mutations are neutral or passenger mutations with little or no deleterious effect, or are low‐frequency constitutional variants within the population [13]. It is important to note that some mutations are specific to particular cancer settings (Figure 3), indicating a potentially tumor‐specific role. However, the functionality of these mutations remains unknown.

### 2.2 Constitutional Genetic Variants in *TET2*

Large genome‐wide association studies (GWAS) have reported associations between high‐frequency genomic variants localizing to the *TET2* gene and the risk of several human cancers, including melanoma [14], breast cancer [15, 16], colorectal cancer [17, 18, 19], endometrial cancer [18] and prostate cancer (PCa) [20, 21, 22, 23, 24]. However, direct evidence supporting a role for the attenuation of TET2 function underpinning these associations is currently lacking, and further work is warranted. Moreover, a recent GWAS incorporating 4018 cases and 10,488 controls did not find any evidence of association for common *TET2* genetic variants and the risk of developing AML [25]. Likewise, GWAS in other myeloid conditions failed to find any evidence for common *TET2* variants significantly associated with disease risk [26, 27].

Rare lowfrequency constitutional variants in *TET2* have also been investigated as risk factors for human cancer. Stremenova‐Spegarova and colleagues [28] investigated three cases of childhood immunodeficiency with concomitant lymphoma and identified rare biallelic *TET2* variants in all three patients. Rare constitutional *TET2* variants have been reported to segregate with hematopoietic malignancy, including lymphoma [29] and myeloid malignancy [30]. Using whole‐exome sequencing, Koboldt et al. [31] reported a significantly higher frequency of rare *TET2* variants in patients with clinically relevant PCa compared to controls (24.4% vs. 9.6%). Taken together, these data provide evidence that rare constitutional genetic variants can predispose to AML and lymphoma and also suggest a role in determining PCa risk.

### 2.3 *TET2* Mutations in Hematological Malignancies

#### 2.3.1 Clonal Hematopoiesis of Indeterminate Potential

Acquired somatic *TET2* mutations in the bone marrow are found in 5%–10% of healthy adults over 65 years of age, imparting a hematopoietic stem cell advantage and subsequent clonal hematopoiesis of indeterminate potential (CHIP), a condition associated with increased risk of myeloid disease [32]. Many mutations observed in CHIP occur in genes not typically associated with cancer development [33] and can predispose individuals to other age‐related conditions such as cardiovascular disease and ischemic stroke [34]. Chou et al. [35] reported *TET2* mutations in 13.2% of their cohort of 486 individuals, with 42.2% having more than one *TET2* mutation, and 6 displaying biallelic mutations. Individuals in this study with *TET2* mutations were significantly older than those without, suggesting the acquisition of *TET2*‐mutant CHIP is age‐associated. A 2012 study [36] found evidence of *TET2* somatic mutations in 10 out of 182 females over 65 years of age, but no mutations were found in those 60 years of age or less. Clinical follow‐up data was obtained for 7 out of these 10 individuals, one of whom later presented with *JAK2* V617F‐positive essential thrombocythemia. Hirsch et al. [37] performed a meta‐analysis of six large CHIP studies, including data from over 4000 patients with a mean cohort age of 59 years. From these data, they concluded that 11%–15% of CHIP is directly related to *TET2* mutations and estimate that approximately 1% of these evolve into MDS or other myeloid disorders. These data support the theory that acquired *TET2* somatic mutations confer age‐related myeloid lineage bias and can predispose individuals to myeloid conditions such as MDS, MPN, and AML. As *TET2* mutations are frequently detectable in multipotent hematopoietic stem cells, it is not surprising that patients with myeloid and lymphoid cancers frequently share a clonal origin [38, 39].

#### 2.3.2 TET2 Mutations in Myeloid Disease

A series of publications in 2009 [40, 41] first reported acquired somatic *TET2* mutations in myeloid disease, including myelodysplastic syndrome (MDS), myeloproliferative neoplasm (MPN), and myeloid malignancies. Although the function of TET2 was unknown at this time, subsequent work has demonstrated a loss of function associated with *TET2* mutation, indicating a role as a tumor suppressor and driver of hematological malignancy. *TET2* mutations were subsequently identified in CD34‐positive progenitor cells in patients with MDS and chronic myelomonocytic leukemia (CMML), suggesting an early clonal origin [42].

Several TET2 residues are recurrently mutated in human myeloid disease, providing evidence for functionality and a role in disease pathogenesis (Table 1, Figure 3). Specifically, mutations at 12 residues in TET2 that co‐occur in solid tumors are reported in myeloid malignancies no < 4 times, with mutations at residues 544, 550, 1380, and 1516 being particularly recurrent in myeloid disease (Table 1, Figure 3) [16]. Likewise, mutations at 19 residues in TET2 are reported at least twice in solid human cancers, which are also reported in myeloid malignancies, with mutations at residues 29, 550, 1516, 1718, and 1762 occurring most frequently in solid tumors (Table 1, Figure 3). Of the high‐frequency recurrent TET2 mutations reported in human cancers, the majority are nonsense variants, with residues 544, 550, and 1516 being particularly affected [16].

**TABLE 1:** Recurrent *TET2* somatic mutations in myeloid malignancy also reported in solid tumors.

TET2 mutation	Reports in solid tumors	Primary tissue	Histology	Tumor‐specific reports	References
R544*	4	Endometrium	Endometrioid carcinoma	2	[43]
		Colon	Adenocarcinoma	1	[44]
		Skin	Malignant melanoma	1	[45]
R550*	11	CNS	Astrocytoma grade IV	1	[46]
		Endometrium	Clear cell carcinoma	4	[43]
		Endometrium	Endometrioid carcinoma	2	[12]
		Colon	Adenocarcinoma	2	[47]
		Not specified	Carcinoma (unspecified)	1	[12]
		Upper aerodigestive tract	Squamous cell carcinoma	1	[12]
R1214Q	2	Rectum	Adenocarcinoma	2	[43, 48]
R1214W	3	Oesophagus	Squamous cell carcinoma	1	[49]
		Colon	Adenocarcinoma	1	[50]
		Skin	Squamous cell carcinoma	1	[43]
H1380Y	3	Breast	Carcinoma (unspecified)	1	[51]
		Lung	Non‐small cell carcinoma	1	[52]
		Oesophagus	Squamous cell carcinoma	1	[53]
R1516*	5	Salivary gland	Salivary duct carcinoma	1	[54]
		Endometrium	Clear cell carcinoma	1	[43]
		Colon	Carcinoma in situ	1	[55]
		Lung	Non‐small cell carcinoma	1	[52]
		Larynx	Squamous cell carcinoma	1	[43]
V1718L	7	CNS	Oligoastrocytoma	3	[56]
		Endometrium	Adenosarcoma	2	[57]
		Skin	Malignant melanoma	1	[58]
		Unspecified soft tissue	Sarcoma	1	[12]
S1898F	2	Lung	Non‐small cell carcinoma	1	[59]
		Upper aerodigestive tract	Carcinoma (unspecified)	1	[52]
P29R	6	Colon	Adenocarcinoma	4	[60]
		Lung	Squamous cell carcinoma	1	[61, 62]
		Thymus	Thymoma	1	[63]
L34F	3	Lung	Adenocarcinoma	2	[64]
		Skin	Basal cell carcinoma	1	[65]
I1762V	33	Colon	Adenocarcinoma	1	[12]
		Lung	Hyperplasia	3	[66]
		Penis	Squamous cell carcinoma	4	[67]
		Prostate	Adenocarcinoma	5	[12]
		Thyroid	Neoplasm	1	[12]
		Pharynx	Nasopharyngeal carcinoma	19	[12]

*Note:* Mutations reported in myeloid malignancies also reported in solid tumors (*n* ≥ 7) are displayed in the top panel. Mutations reported in solid tumors also reported in myeloid malignancies (*n* ≥ 3) are displayed in the lower panel. Data gathered from the COSMIC GRCh37 v96 database [8]. * ‐ stop codon.

#### 2.3.3 TET2 Mutations in Lymphoma and Lymphoid Leukemia

**FIGURE 4:**
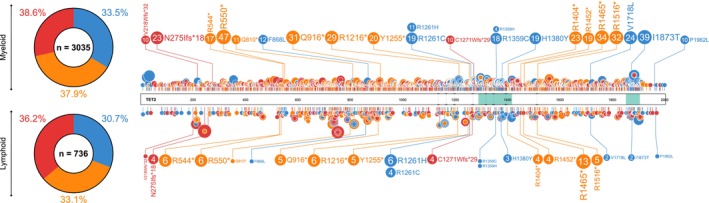
Distribution of missense, nonsense, and frameshift somatic mutations reported in TET2 for lymphoid malignancies. Green areas indicate the location of oxygenase domains on TET2 protein, and dashed lines represent the intersection between exons (exons 3–11). Blue, missense mutation; orange, nonsense mutation; red, frameshift mutation. Noncoding mutations, untranslated region (UTR) mutations, and in‐frame deletions/insertions are not shown. Donut plots highlight the percentages of each mutation type for myeloid malignancies (*n* = 3035) and lymphoid malignancies (*n* = 736). Mutations reported in the Catalogue of somatic mutations in Cancer (COSMIC) GRCh37.COSMIC v96 database [8] in myeloid malignancies (top) and lymphoid malignancies (bottom) are shown in lollipop plot. Only mutations recurrently reported across myeloid and lymphoid settings are highlighted (*n* > 10 in either myeloid or lymphoid, with *n* ≥ 1 of the same mutation in the other setting). Circles indicate the amino acid residue location of the mutation, and circle size corresponds to the number of reported mutations at each site. Lollipop plots were created using ProteinPaint web tool developed by St. Jude Children's Research Hospital prior to final figure configuration.

There are over 900 reports of somatic *TET2* mutations in lymphoid neoplasms in COSMIC, with missense, nonsense, and frameshift mutations accounting for 736. A proportion of these mutations is also reported in myeloid disease (Figure 4) [12], implicating them as functional mutations in both settings.

Loss of 5hmC is a common finding in B‐cell lymphomas. Tanager et al. [68] documented a reduction in 5hmC in the neoplastic cells of the majority of low‐grade and high‐grade B‐cell lymphoma, as well as Hodgkin's lymphoma (HL) (94%, 94%, and 89% of cases, respectively) (*n* = 92). However, in diffuse large B‐cell lymphoma (DLBCL), Tanager and colleagues confirmed that although most tumors showed a reduction in 5hmC, only approximately half harboured mutations in epigenetic regulators such as *TET2* and *DNMT3A*. Likewise, Lemonnier et al. [69] demonstrated that 5hmC loss was independent of *TET2* or *DNMT3A* mutation in approximately 50% of peripheral T‐cell lymphomas.

Mouse models have demonstrated that *TET2* deficiency predisposes individuals to both T‐cell and B‐cell malignancies, although additional co‐operating mutations are required for malignant transformation [70]. *TET2* mutations are observed in a larger proportion of T‐cell compared to B‐cell lymphomas [71, 72], loss of function *TET2* mutations have been described in both [73, 74], suggesting a broad lineage‐independent tumor suppressor role for *TET2* in lymphomagenesis. As mutations involving *TET2* primarily occur very early in the hematopoietic lineage [73], it is not surprising that *TET2* mutations are drivers of lymphoid as well as myeloid disease.

Fraietta and colleagues [75] describe findings from a 78‐year‐old male with relapsed/refractory chronic lymphocytic leukemia (CLL) treated with chimeric antigen receptor (CAR) T‐cells targeting the CD19 protein. At the peak of clinical activity, it was discovered that the clonal expansion of CAR T‐cells in this patient had arisen primarily from a single cell clone with a biallelic *TET2* mutation, exhibiting a hypomorphic E1879Q variant encoded by one *TET2* allele, and a compound heterozygous loss‐of‐function mutation in the other. Fraietta et al. investigated this further, using in vitro re‐stimulation assays to assess the effect of TET2 inhibition on CAR T‐cell function. Results revealed that repeated stimulation with CD19^+^ tumor cells allowed continual expansion of *TET2* knockdown CAR T‐cells in an antigen‐dependent manner, whereas this same re‐stimulation in CAR T‐cells with wildtype *TET2* resulted in complete arrest of cell growth. In this study, TET2 dysfunction was further shown to produce CAR T‐cells displaying properties of both short‐and long‐lived memory cells, able to multiply and provoke effector responses. These findings suggest that targeting the epigenome through genes such as *TET2* may shape the immune response and improve the efficacy of CAR T‐cell therapy in CLL.

### 2.4 *TET2* ‐Mutant Allele Dosage in Human Cancer

**FIGURE 5:**
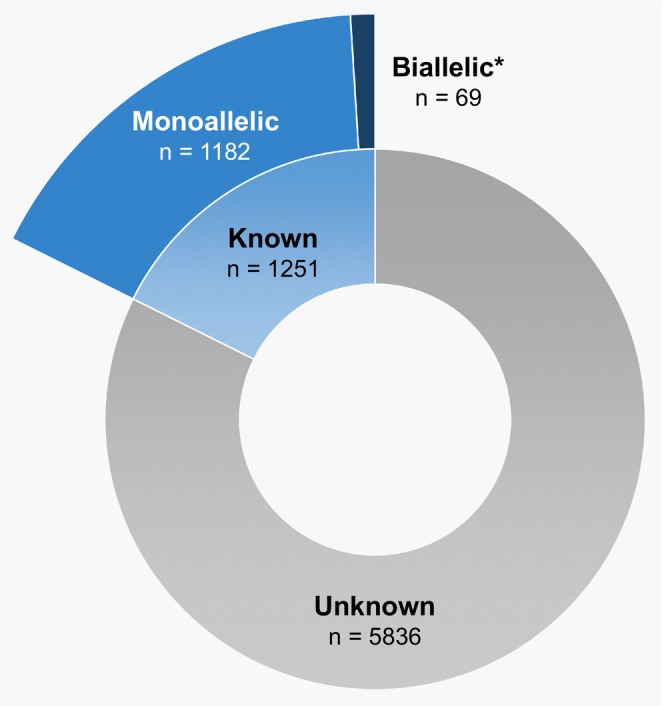
Number of reported monoallelic and biallelic *TET2* mutations in cancer. Of a total 7087 *TET2* mutations reported in the Catalogue of somatic Mutations in Cancer (COSMIC) GRCh37.COSMICv96 database, mutant‐allele dosage is reported for only 17.6% (*n* = 1251). Of these, only 5.5% (*n* = 69) are reported as biallelic [8]. *Data in this figure are solely based on reports from COSMIC; therefore, some double mutations may be incorrectly reported as biallelic as the allelic origin of these mutations is not confirmed. Also includes compound heterozygous mutations.

Despite numerous reports of somatic *TET2* mutations in human cancer, mutant‐allele dosage remains underreported (Figure 3), in part due to the complex nature of biallelic mutations, which often involve compound heterozygosity, and the technical challenge of discerning whether multiple mutations are present on the same allele. It is hypothesized that biallelic *TET2* mutations provide a competitive advantage to cells over monoallelic mutations [76]; however, determining the consequences of monoallelic or biallelic loss of function on overall gene expression in individual tissue samples is technically challenging [77]. Furthermore, data concerning the effects of *TET2*‐mutant allele dosage in cancer development is scarce (Figure 5), and relatively few studies have sought to differentiate between monoallelic and biallelic *TET2* mutations in humans.

**FIGURE 6:**
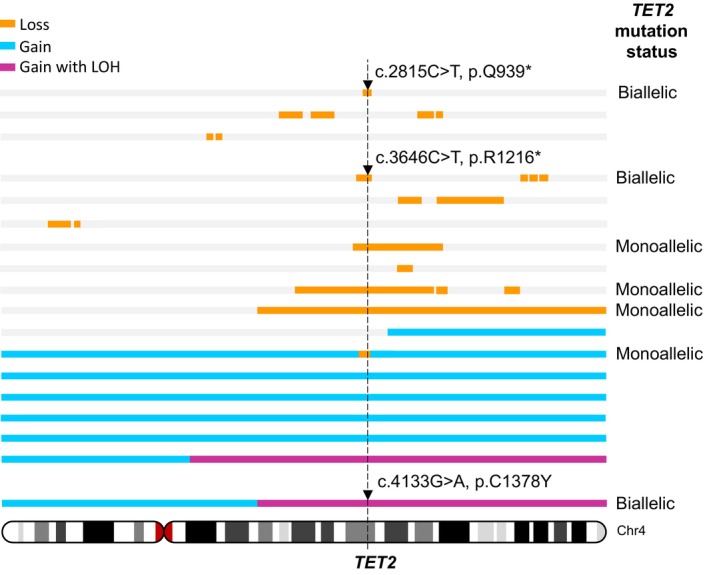
Somatic mutations affecting the TET2 locus in AML patients with cytogenetic abnormality of chromosome 4. Depicted are regions of copy number gain (blue), gain with concomitant loss of heterozygosity (LOH) (purple), and loss (orange) affecting chromosome 4 discerned using high‐density single nucleotide polymorphism (SNP) arrays in 18 AML patients with cytogenetically detectable chromosome 4 abnormalities. Base substitution mutations (indicated by black triangles) were determined using *TET2* exon sequencing. The dashed vertical line indicates the approximate location of *TET2* on chromosome 4. Adapted from [35].

Stölzel et al. [3] described an elderly AML patient with a somatic *TET2* mutation. Sequencing analysis of the leukemic blasts at both diagnosis and relapse indicated disease pathogenesis was initiated by a *TET2* nonsense base substitution mutation in exon 3 with a focal 1.1 Mb deletion in the remaining *TET2* allele, followed by the acquisition of an *NPM1* mutation, giving rise to leukemia. The *TET2* deletion and mutation remained detectable at high levels in remission bone marrow samples, despite the bone marrow appearing morphologically normal. These data indicate that in this case *TET2* mutation alone was not sufficient to result in malignant transformation, but strongly implicates it as a leukemic driver. Stölzel and colleagues used exome sequencing and high‐density polymorphism arrays to characterize *TET2* mutations in a series of 30 AML cases with cytogenetically visible chromosome 4 abnormalities (Figure 6). This analysis revealed several cases with whole chromosome 4 trisomy, which included one case with a focal deletion affecting the *TET2* locus in one of the three alleles. Two of the cases with trisomy 4 also had homozygosity across most of the long arm of the chromosome (affecting *TET2*), one of which carried a base substitution mutation in all three alleles. Although Stölzel and colleagues did not identify any cases with monosomy 4 by polymorphism array, there were nine cases with interstitial deletions and one with a telomeric deletion. Of these, the *TET2* locus was affected by the deletion in five patients, and two of these also carried a base substitution in the remaining allele.

As such, in a panel of 30 AML cases with a cytogenetically discernible chromosome 4 abnormality, seven patients (23%) had loss of function *TET2* mutations, including 4 (13%) with monoallelic *TET2* mutation (all of which were whole gene deletions resulting in reduced *TET2* copy number). Three patients (10%) carried biallelic *TET2* mutation; whole gene deletion plus nonsense mutation in two cases and biallelic base substitution resulting from a whole chromosome gain and loss of heterozygosity (LOH) in the third case. In addition, further three cases had aneuploidies (two with trisomy and one with monosomy of chromosome 4) in low‐frequency subclones identified cytogenetically, but which were not detectable using polymorphism arrays (Figure 6). These data demonstrate that *TET2* alterations are complex, involving base substitution mutations often in combination with gains and losses of whole or partial chromosome 4, small deletions, and acquired copy number LOH.

Studies carried out in mice investigating the relationship between *TET2*‐mutant allele dosage and myeloid disorders have yielded interesting results. Li and colleagues [78] developed an in vivo *TET2* knockout mouse model comparing the development of myeloid malignancy between *TET2* ^+/−^ (*n* = 66) and *TET2* ^−/−^ (*n* = 62) mice. TET2 knockout (*TET2* ^−/−^) mice showed a significant increase in global genomic levels of 5mC and a concomitant reduction of 5hmC in DNA from the bone marrow, compared to *TET2* ^+/−^ counterparts. This study also established a correlation between *TET2* status and myeloid malignancy‐related death, with approximately 33% (21/62) of *TET2* ^−/−^ mice dying before 1 year of age with death directly linked to myeloid malignancy, compared to only 8% (5/66) of *TET2* ^+/−^ mice. Prior to disease onset, *TET2* ^−/−^ mice displayed an increased pool of Lin‐Sca‐1^+^c‐Kit^+^ hematopoietic multipotent stem cells, leading to a discernible skewing toward monocytic and granulocytic cell lineages. Similar observations were found in a study by Moran‐Crusio et al. [39], where loss of *TET2* was shown to increase stem‐cell self‐renewal in vivo, ultimately leading to the development of myeloproliferative disorders accompanied by splenomegaly, extramedullary hematopoiesis, and monocytosis.

## 3 *TET2* Mutations in Solid Tumors

**FIGURE 7:**
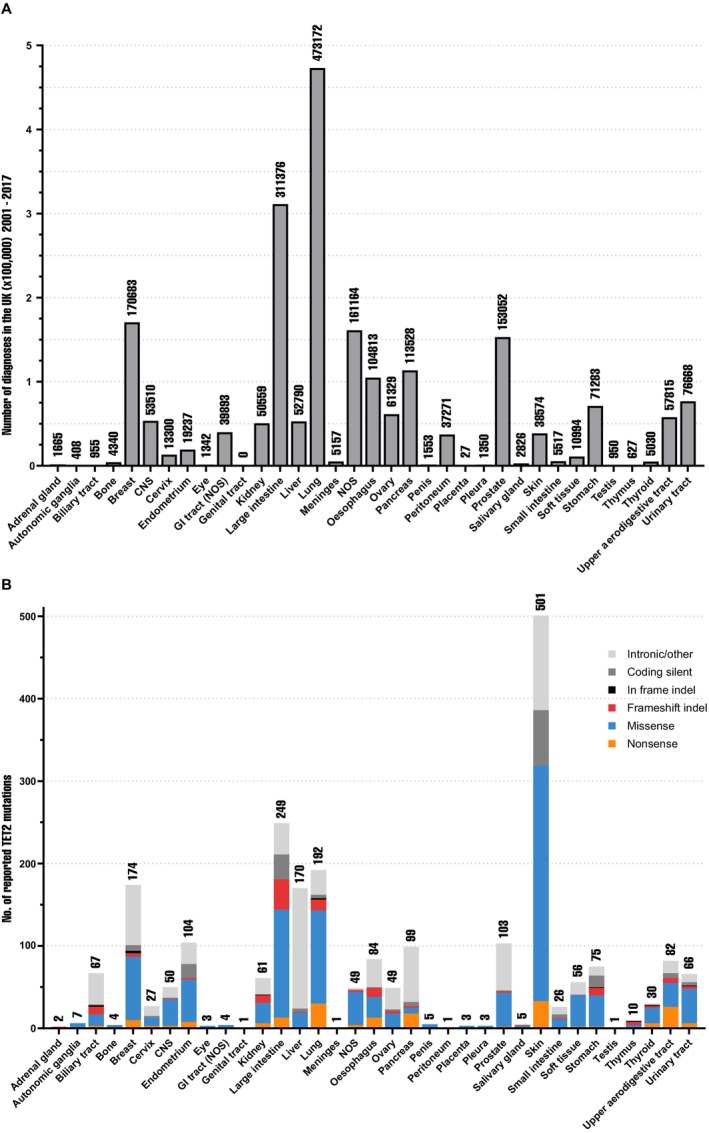
Frequency of solid tumors and total number and type of reported *TET2* mutations. (A) Total number of solid tumor diagnoses from 2001 to 2017 in the United Kingdom by primary tissue [38]. (B) Frequency of nonsense (orange), missense (blue), frameshift indel (red), coding silent (dark gray), in‐frame indel (black), and unknown (light gray) TET2 mutations in solid tumors. Data collected from the Catalogue of Somatic Mutations in Cancer (COSMIC) GRCh7.COSMIC v96 variant mutation tables by tumor type [8]. Primary tissues with no reported TET2 mutations in COSMIC have not been included in this figure.

*TET2* mutations are reported in the majority of human solid tumors at varying frequencies [12]. Tumors arising from the primary tissue of the breast, lung, large intestine, and prostate occur at a substantially higher incidence than other sites [79] (Figure 7A); thus, it is not surprising that these tumors harbour some of the highest numbers of reported somatic *TET2* mutations (Figure 7B). There are over 2000 total reported cases of *TET2* mutation in solid tumors in COSMIC, of which *TET2*‐mutant breast, lung, large intestine, and prostate constitute 30.4% (718/2364) [12].

*TET2* mutation is associated with solid tumor development and progression, but these relationships differ for malignancies arising from different primary tissues. Although *TET2* mutation is primarily associated with early‐stage cancer development, there are reports of *TET2* mutation specific to metastatic disease in some solid tumors. For example, Nguyen et al. [80] describe a mouse model in which *TET2* mutated tumor infiltrating lymphocytes (TILs) derived from clonal hematopoiesis stimulate tumor angiogenesis in lung cancer, exacerbating tumorigenesis and promoting metastasis. TET2 knockdown has also been shown to markedly enhance the migratory, invasive, and proliferative properties of lung adenocarcinoma cells in vitro through activation of the cGAS‐STING signaling pathway [81]. Reduced TET2 expression is also associated with lymph node metastasis in ovarian serous cystadenocarcinoma [82], and both local and distant metastasis in PCa [12, 83].

**FIGURE 8:**
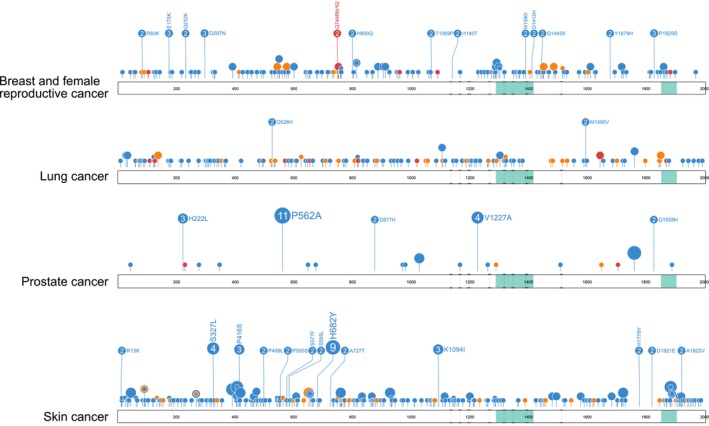
Recurrent tumor‐specific TET2 mutations are present across a range of common cancers. Extended and annotated circles represent recurrent (*n* ≥ 2) tumor‐specific mutations reported in breast and female reproductive cancers, lung cancer, prostate cancer, and skin cancer that are not reported in any other malignancy in the Catalogue of somatic mutations in Cancer (COSMIC) GRCh37.COSMIC v96 database [8]. Green areas indicate the location of oxygenase domains on TET2 protein, and dashed lines represent the intersection between exons (exons 3–11). Blue, missense mutation; orange, nonsense mutation; red, frameshift mutation. Noncoding mutations, untranslated region (UTR) mutations, and in‐frame deletions/insertions are not shown. Circles indicate the amino acid residue location of the mutation, and circle size corresponds to the number of reported mutations at each site. Lollipop plots were created using ProteinPaint web tool developed by St. Jude Children's Research Hospital prior to final figure configuration.

*TET2* has been implicated as a tumor suppressor gene in various epithelial tissues. Numerous recurrent *TET2* somatic mutations seen in myeloid malignancy are also reported in solid tumors of several primary tissues (Figure 3), suggesting functionality and a role in pathogenesis in these tumors. For example, the R550* nonsense mutation is the most frequent *TET2* somatic mutation reported in myeloid malignancy (47/3035) [40] and is also reported in lymphoma [12], astrocytoma [46], colorectal adenocarcinoma [47], endometrioid clear cell carcinoma [43], and squamous cell carcinoma of the upper aerodigestive tract [12] (Table 1). Although many *TET2* mutations are recurrent across a range of malignancies, there are a number of mutations that appear to be tumor‐specific [12]. For example, recurrent (*n* ≥ 2) tumor‐specific mutations reported in COSMIC for breast and female cancers account for 15.5% of total mutations (only including missense, nonsense and frameshift mutations) (29/187), 2.4% in lung cancer (4/162), 52.3% in PCa (22/42) and 14.7% in skin cancer (37/252) (Figure 8), as well as in other settings such as liver and kidney. The functionality of these mutations remains largely unknown, and further research is warranted.

It is important to note that somatic mutations are not always functional and may have little to no effect on TET2 mRNA transcript expression, TET2 protein, or genomic methylation [84]. As such, it should be recognized that these are not established surrogate markers for *TET2* mutation.

### 3.1 Clonal Hematopoiesis in Solid Tumors

CHIP occurs in patients with solid tumors as well as in those with myeloid malignancies. Although more commonly observed in the latter, the incidence is significantly higher in individuals with solid tumors than in the general ageing population [85]. Coombs et al. [85] established that out of 5649 patients with underlying CHIP, 14% had recurrent mutations in genes involved in epigenetic pathways. *TET2* mutations (*n* = 205) primarily resulted in protein truncations similar to those seen in myeloid disease.

Coombs and colleagues also sought to investigate any apparent relationship between the incidence of CHIP and solid tumors by location. Patients diagnosed with thyroid cancer presented with the highest incidence of CHIP (40%), and the lowest frequency of CHIP was observed in individuals with germ cell tumors. Furthermore, having received prior radiation therapy was significantly correlated with CHIP (*p* < 0.001), as was previous tobacco use (*p* < 0.001). Next‐generation sequencing on 8810 individuals with various solid cancers revealed clonal hematopoiesis in up to 25% of patients, 4.5% of whom harbored mutations in leukemia driver genes. TILs harboring mutations associated with CHIP are found in as many as 50% of primary breast cancers [86] and are often enriched in breast cancer tissues before the detection of CHIP in the blood [87]. These data suggest that CHIP affects solid tumor development and progression, presumably by modulating immune function in the microenvironment [80]. CHIP associated with the *TET2* mutation is also linked to an increased risk of other conditions, such as cardiovascular disease, likely via effects on the local microenvironment [34, 88].

When *TET2* mutation is detected in nonhematological tumor samples, clonal hematopoiesis can be difficult to differentiate from tumor‐specific somatic mutations. Without a matched peripheral blood sample, TILs possessing CHIP‐related mutations detected within a tumor sample could lead to erroneous calling of tissue‐specific tumor variants [89], potentially leading to recommendations for inappropriate targeted therapies. If both tumor and peripheral blood have been sequenced, a higher variant allele frequency (VAF) from the blood than the tumor indicates the detected clone is likely of hematopoietic origin and therefore should not be involved in considering treatment strategies [33].

### 3.2 Prostate Cancer

Loss of TET2 is a potentially informative biomarker of PCa progression, where the acquisition of somatic *TET2* mutations is associated with an increased risk of metastatic disease [83]. A series of studies by Nickerson et al. [83, 90] revealed that the number of somatic *TET2* mutations is significantly greater in metastatic disease compared to primary tumors. In one such study [83], *TET2* was altered in all metastatic samples (*n* = 11) via a somatic C>G missense mutation encoding a nonconservative alanine to proline substitution (p.P562A), which was not observed in any of the matched primary tumors. Interestingly, the p.P562A mutation appears to be specific to PCa, with no reports of this mutation in any other cancer type in COSMIC [12]. Furthermore, Nickerson and colleagues [83] analyzed next‐generation sequencing data from cBioPortal and reported significantly more *TET2* somatic alterations in metastatic PCa (23/117, 19.7%) compared to primary tumors (14/246, 5.7%) (*p* < 0.001).

*TET2* is involved in androgen receptor (AR)‐mediated signaling [83], with AR‐mediated induction of the miR‐29 family directly targeting *TET2*, resulting in downstream activation of integral pathways involved in PCa development. Kamdar et al. [91] sought to determine the relationship between *TET2* loss and PCa progression and reported seven genes downregulated in a *TET2* knockout cell line model: *ASB2*, *ETNK2*, *MEIS2*, *NRG1*, *NTN1*, *NUDT10*, and *SRPX*. Kamdar and colleagues performed survival analysis of a PCa patient cohort taken from The Cancer Genome Atlas (TCGA) and showed that reduction in *ASB2*, *NUDT10*, and *SRPX* expression was significantly associated with lower recurrence‐free survival. Furthermore, *ASB2*, *MEIS2*, and *SRPX* had significantly lower expression in high‐risk tumors compared to intermediate or low risk, suggesting these genes could be crucial indicators of PCa progression. Thus, it is also important to look past *TET2* attenuation and understand the downstream effects, as it is evident that different downstream targets are linked to disease progression and patient outcomes.

Taken together, these data suggest that *TET2* mutation associates with late‐stage disease and disease progression in a subset of PCa patients, rather than being an initiating event as reported in myeloid disease. *TET2* is an established tumor suppressor gene [92], although an oncogenic function in the context of PCa cannot be excluded.

### 3.3 Skin Cancer

5hmC loss is an epigenetic hallmark of melanoma [93, 94]; however, it is unclear whether this directly contributes to melanomagenesis or rather results from the epigenetic remodelling observed in malignant transformation. Data suggest melanoma progression is accompanied by complete remodelling of the hydroxymethylation landscape, involving an overall decrease in 5hmC levels but with gain at discrete loci. A similar phenomenon is also observed in oral, oesophageal, and cutaneous squamous cell carcinoma (SCC) [93, 95, 96]. This reduction in genomic 5hmC levels has been directly linked to reduced TET activity. Bonvin et al. [95] revealed in a murine study that loss of one *Tet2* allele accelerates melanomagenesis, implicating *Tet2* as a haploinsufficient tumor suppressor gene in mice. However, mice with a germline Tet2 null genotype (*Tet2* ^−/−^) show no evidence of increased sporadic tumor formation, with *NRAS* ^Q61K^ mutation required for malignant melanoma development [93, 95].

Most melanoma driver genes are not regulated by methylation, although it is hypothesized that *TET2* has additional effects on other mechanisms such as genomic instability and DNA damage repair [95]. In cutaneous SCC, genomic alterations are found in *TET* genes at a frequency of approximately 3%–4%, with most cases carrying alterations in more than one *TET* gene concomitantly [93]. Likewise, Bonvin and colleagues [95] found a majority of melanoma samples in their study carried mutations in another *TET* gene in addition to *TET2*. Together, these data suggest that the loss of TET2 function is insufficient for malignant transformation in skin cancers and imply that the concomitant loss of TET1 or TET3 may be required for melanomagenesis [3].

### 3.4 Lung Cancer

The direct mechanism by which TET2 loss contributes to lung cancer progression is unknown; however, *TET2* is recognized as a tumor suppressor in non‐small‐cell lung cancer (NSCLC) [81]. There is also evidence to suggest that a reduction in TET2 protein results in increased angiogenesis in lung adenocarcinoma tumor samples through upregulation of *VEGFa* transcript, allowing for increased tumor growth and thus driving aggressive disease [80].

Xu et al. [97] sought to address the involvement and impact of *TET* mutations in lung adenocarcinoma using various TCGA and cBioPortal datasets (*n* = 304). Mutations in the *TET* genes were reported in approximately 7.4% of patients, with mutations in *TET1*, *TET2*, and *TET3* present in 4%, 1.6%, and 1.8%, respectively. The majority of mutations reported in the *TET* genes were missense (83%), and the remainder were truncating. Interestingly, almost a third (32%) of *TET*‐mutated lung adenocarcinomas in these datasets carried an additional mutation in *KRAS*. Xu and colleagues demonstrated that patients with *TET* mutations had a significantly reduced survival outcome if they also presented with a *KRAS* mutation (*p* ≤ 0.01), compared to patients who harbored a *TET* mutation alone. Specifically, patients with concurrent *TET2*/*KRAS* mutations (*n* = 5) had a significantly worse outcome than patients with independent *TET2* mutation (*n* = 8, *p* ≤ 0.05) or *KRAS* mutation (*n* = 263, *p* ≤ 0.05). Although these results show significance, it should be recognized that this study is limited by small case numbers across some of the study arms.

Although there is a clear role for TET2 loss in the development and progression of some lung cancers, considerably more research is required in this area to fully elucidate the impact of *TET2* mutations in this setting.

### 3.5 Breast and Female Reproductive Cancers

Oestrogen receptor (ER)‐positive breast cancer cells have an inherently high level of the transcription factor GATA binding protein 3 (GATA3). Loss of GATA3 from the ER complex in ER‐positive breast cancer cells is associated with a reduction in the expression of TET2; thus, it is postulated that TET2 expression is reliant on GATA3 in this setting [98]. Furthermore, TET2 and ER share downstream regulatory targets across the genome [98]. TET2 is crucial for the appropriate expression of both ER and GATA3 target genes, indicating a functional role of TET2 in mediating ER –chromatin interactions. Attenuation of TET2 is also linked to malignant breast tumor development [82, 99]. In vitro studies revealed that cell migration and invasion are inhibited in breast cancer cells null for the oncogene lysine‐specific demethylase 2A (KDM2A), via TET2‐mediated activation of the downstream tumor suppressors E‐cadherin and epithelial cell adhesion molecule (EpCAM) [99]. Likewise, TET2 knockdown decreases E‐cadherin and EpCAM levels and is correlated with increased tumor invasiveness [99].

Laurent et al. [100] demonstrated that overexpression of TET2 reduced the tumorigenic potential of MCF‐7 breast cancer cells and increased the rate of cell death in vitro. This is consistent with other studies showing that both TET1 and TET2 expression leads to reduced tumor growth in xenograft mouse models [94, 101]. Laurent and colleagues [100] also analyzed gene expression data from a cohort of breast cancer patients and revealed that both TET1 and TET2 expression significantly decreased with tumor progression (*p* < 0.0001). Conversely, *TET3* mRNA transcript levels were greater in tumor tissue and positively correlated with tumor progression.

Based on data from TCGA, Wan et al. [82] established that *TET2* mutation was reported at high frequency in the following subsets of female cancers: Breast invasive carcinoma (BICA), cervical squamous cell carcinoma and endocervical adenocarcinoma (CESC), ovarian cystadenocarcinoma (OV), uterine corpus endometrial carcinoma (UCEC), and uterine carcinosarcoma (UCS). Mutations at residue 1516 were the most common, with multiple reports of the nonsense mutation R1516* or the missense mutation R1516Q in UCEC, CESC, and BICA. R1516* mutation is also reported in myeloid disease, salivary duct carcinoma, colon adenocarcinoma in situ, NSCLC, and squamous cell carcinoma of the larynx (Table 1, Figure 3) [12], implicating this mutation as a driver of tumorigenesis.

### 3.6 Other Solid Tumors

Genetic alterations in *TET2* are frequent in numerous cancer settings, although data regarding the biological consequences of TET2 loss in nonhematological malignancies is limited. Mutations in other *TET* genes are also observed, and TET1 loss has been implicated as a driver of tumorigenesis in colorectal cancer (CRC) via inhibition of the WNT pathway [102]. TET2 loss has been shown to increase methylation at chemokine gene promoter regions in CRC [103], which alongside loss of nuclear TET2 localization correlates with a more aggressive metastatic phenotype [104].

Data surrounding the TETs are particularly limited in rarer cancer types; however, somatic *TET2* mutations have been described in a number of these settings, including salivary duct carcinoma [54], penile squamous cell carcinoma [105], parathyroid carcinoma [106], and oligoastrocytoma [56]. Using a cell line derived from a hyperplastic parathyroid tumor (sHPT‐1), Barazeghi and colleagues [106] demonstrated that *TET2* knockout significantly increased cell colony growth in vitro. Further experiments also showed an increase in migration velocity, together supporting the function of TET2 as a tumor suppressor gene in parathyroid tissues.

In thyroid neoplasia, reduced mRNA transcript levels of *TET1* and *TET2*, coupled with increased mRNA levels of *DNMT1*, drive DNA hypermethylation of promoter regions of the *PTEN* and *CDKN2A* tumor suppressor genes (TSGs) [10]. The level of promoter methylation at these TSGs has been linked to the aggressiveness and progression of several types of thyroid cancer, including papillary thyroid carcinoma and follicular thyroid carcinoma [107, 108]. *TET2* loss is also associated with the development and progression of thymic carcinoma. Saito et al. [9] performed genome‐wide methylation analysis of *TET2* mutation‐positive (*n* = 3) and *TET2* mutation‐negative (*n* = 7) thymic carcinoma samples and described a higher degree of methylation at discrete loci in *TET2* mutation‐positive tumors compared to those that were *TET2* mutation‐negative.

Although this review focuses on *TET2* mutation, there is evidence that mutations in other critical genes can also give rise to a global genomic hypermethylation phenotype in both hematological and nonhematological cancers, including *WT1* in AML [109] and *IDH1*/*2* in AML [110] and glioma [111].

## 4 Prognostic Impact of *TET2* Mutations in Cancer

### 4.1 Prognostic Impact of *TET2* Mutation in Hematological Malignancies

A number of studies show a lack of association between *TET2* loss of function mutation and OS in MDS [92, 112, 113]. However, in a meta‐analysis comprising 16 AML studies [114], *TET2* mutation was associated with a significant adverse impact on both OS (HR [95% CI] = 1.386 [1.217–1.577], *p* < 0.001) (Table 2) and event free survival (EFS) (HR [95% CI] = 1.594 [1.187–2.141], *p* = 0.002). Specifically, *TET2* mutation was associated with an unfavorable OS in AML patients under 65 years of age (HR [95% CI] = 1.310 [0.999–1.718], *p* = 0.051) and in patients with normal cytogenetics (HR [95% CI] = 2.034 [1.440–2.872], *p* < 0.001) or intermediate risk cytogenetics (HR [95% CI] = 1.662 [1.312–2.105], *p* < 0.001). Likewise, Chou et al. [35] reported lower OS for AML patients with intermediate risk cytogenetics carrying a mutation in *TET2* (HR [95% CI] = 1.804 [0.934–3.484]).

**TABLE 2:** Patient outcome related to low *TET2* expression in hematological disorders.

Hematological disorder	HR	95% CI	*p*	Outcome	*n*	First author, year	Ref
ALL	**3.115**	**1.42–1.61**	**0.005**	Unfavorable OS	130	Zhang, 2019	[115]
MDS	1.32	0.99–1.94	0.122	ns	78	Lee, 2022	[113]
AML^a^	**1.386**	**1.22–1.58**	**< 0.001**	Unfavorable OS	4378	Wang, 2019	[114]
nALCL	0.99	0.4–2.47	0.75	ns	25	De Pádua Covas Lage, 2022	[116]
ALCL	1.01	0.32–3.14	0.99	ns	34	De Pádua Covas Lage, 2022	[116]
PTCL	1.65	0.69–3.94	0.262	Unfavorable OS	46	Ye, 2021	[117]

*Note:* Significant values (*p* < 0.05) are in bold.

Abbreviations: ALCL, anaplastic large cell lymphoma; ALL, acute lymphoblastic leukemia; AML, acute myeloid leukemia; MDS, myelodysplastic syndrome; n/a, not applicable; nALCL, non‐anaplastic large cell lymphoma; ns, not significant; OS, overall survival; PTCL, peripheral T‐cell lymphoma.

^a^ AML data from published meta‐analysis.

Ahn et al. [118] sought to elucidate the prognostic role played by *TET2* mutation in patients with normal karyotype (NK)‐AML (*n* = 407), and particularly in patients with homozygous *TET2* mutation. Multivariate analysis revealed that homozygous *TET2* mutation was not significantly associated with OS (HR [95% CI] = 1.207 [0.799–1.825], *p* = 0.472), but was associated with a markedly increased relapse incidence (RI) compared to wild‐type *TET2* (5 year RI 100% vs. 43.1%, *p* = 0.002), single *TET2* mutation (5 year RI 100% vs. 41.1%, *p* = 0.012) or heterozygous double *TET2* mutation (5 year RI 100% vs. 27.3%, *p* = 0.023). Thus, homozygous *TET2* mutation was deemed an independent adverse prognostic factor for RI (HR [95% CI] = 1.519 [1.105–2.086], *p* < 0.001). These data suggest that mutant‐allele dosage can have a significant effect on patient outcome in AML, and is therefore a crucial factor when considering patient management strategy.

Reduced *TET2* mRNA expression is an adverse prognostic marker in pediatric acute lymphoblastic leukemia (ALL), where low *TET2* transcript in leukemic lymphocytes is associated with a significantly lower 5‐year OS (HR [95% CI] = 3.115 [1.42–6.81], *p* = 0.005) (Table 2) and EFS (HR [95% CI] = 3.49 [1.59–7.69], *p* = 0.002) (*n* = 130) [115]. In a cohort of patients with nodal peripheral T‐cell lymphoma (nPTCL) (*n* = 59), *TET2* mutation was found to have no significant impact on OS (HR [95% CI] = 0.98 [0.689–3.937]) (Table 2) [116]. De Pádua Covas Lage and colleagues [116] stratified nPTCL cases into anaplastic large cell lymphoma (ALCL) (*n* = 34) and non‐ALCL (nALCL) (*n* = 25), in which neither subgroup showed a significant relationship between *TET2* mutation and OS (HR [95% CI] = 1.01 [0.32–3.14], *p* = 0.99, and HR [95% CI] = 0.99 [0.40–2.47], *p* = 0.75, respectively) (Table 2). Ye et al. [117] also report a lack of association between *TET2* mutations and OS (HR [95% CI] = 1.646 [0.689–3.937]) (*p* = 0.262) in peripheral T‐cell lymphoma (PTCL) patients (*n* = 46) (Table 2).

### 4.2 Prognostic Impact of *TET2* Mutation in Solid Tumors

There is considerable data reporting *TET2* mutation/loss and association with survival in female‐associated cancers, such as breast, ovarian, cervical, and endometrial cancers [82, 119, 120]. Multivariate cox regression analysis by Wan et al. [82] demonstrated that low *TET2* mRNA expression was an independent prognostic factor in patients with breast carcinoma (HR [95% CI] = 1.47 [1.11–1.92], *p* = 0.0054) (Table 3). Among breast carcinoma subgroups, low *TET2* expression was associated with poorer OS in estrogen receptor (ER)‐negative (HR [95% CI] = 2 [1.25–3.125], *p* = 0.0027), human epithelial growth factor receptor 2 (HER2)‐negative (HR [95% CI] = 1.59 [1.15–2.17], *p* = 0.0044), progesterone receptor (PR)‐negative (HR [95% CI] = 1.22 [0.64–2.33], *p* = 0.55), grade 1 (HR [95% CI] = 1.37 [0.12–16.6], *p* = 0.8), and grade 3 (HR [95% CI] = 1.72 [1.03–2.86], *p* = 0.025) breast carcinomas, and breast carcinoma with lymph node involvement (HR [95% CI] = 0.47 [0.29–0.77], *p* = 0.0023) (Table 3). Low *TET2* expression was also associated with poor OS in early‐stage breast cancer (HR [95% CI] = 2.59 [0.352–19.172], *p* = 0.067) [119] (Table 3). Wan and colleagues [82] also published data on ovarian carcinoma, with a significant association between low *TET2* transcript levels and shorter OS (HR [95% CI] = 1.26 [1–1.57], *p* = 0.048), particularly in patients with stage 1 or stage 2 disease (HR [95% CI] = 5 [1.14–25], *p* = 0.019) (Table 3). Zhang and colleagues [120] reported that complete loss of TET2 protein in endometrial carcinoma was significantly associated with shorter OS (46.74 months) compared to TET2‐positive cases (66.57 months) (HR [95% CI] = 24.189 [3.115–187.822], *p* < 0.01) [121] (Table 3).

**TABLE 3:** Patient outcome related to low *TET2* mRNA expression in various solid tumors.

Primary tissue	Disease subtype/stage	HR	95% CI	*p*	Outcome	*n*	First author, year	Ref
Breast	Breast invasive carcinoma	**1.47**	**1.11–1.92**	**0.0054**	Unfavorable^a^	1880	Wan, 2022	[82]
	ER+	1.44	0.83–3.97	0.27	ns	221		
	ER—	**2**	**1.25–3.125**	**0.0027**	Unfavorable OS	284		
	PR+	0.49	0.17–1.45	0.19	ns	144		
	PR—	1.22	0.64–2.33	0.55	ns	148		
	HER2+	1.09	0.66–1.79	0.75	Unfavorable OS	223		
	HER2—	**1.59**	**1.15–2.17**	**0.004**	Unfavorable OS	72		
	LN involvement	**2.12**	**1.29–3.45**	**0.0023**	Unfavorable OS	230		
	Grade 1	1.37	0.12–16.6	0.8	ns	26		
	Grade 2	2.38	0.71–7.69	0.150	ns	64		
	Grade 3	**1.72**	**1.03–2.86**	**0.025**	Unfavorable OS	91		
	TP53 mutant	1.04	0.27–4	0.93	ns	56		
Breast	Early breast cancer	2.59	0.35–19.17	0.067	ns	114	Yang, 2015	[119]
Endometrium^b^	Endometrial adenocarcinoma	**24.19**	**3.12–187.82**	**< 0.01**	Unfavorable OS	88	Zhang, 2022	[120]
Head and neck	Squamous cell carcinoma	1.52	0.68–3.33	0.48	ns	117	Huang, 2020	[121]
Head and neck	Squamous cell carcinoma	**1.96**	**1.1–3.78**	**0.044**	Unfavorable OS	101	Misawa, 2019	[122]
Liver	Intrahepatic cholangiocarcinoma	**0.34**	**0.15–0.75**	**0.007**	Favorable OS	52	Yamashita, 2022	[123]
Lung	Non‐small‐cell lung cancer	**0.15**	**0.03–0.90**	**0.038**	Favorable OS	108	Zhao, 2020	[124]
Ovary	Ovarian carcinoma	**1.26**	**1–1.57**	**0.048**	Unfavorable OS	655	Wan, 2022	[82]
	Endometrioid	1.47	0.2–10	0.7	Ns	30		
	Serous	1	0.8–1.25	1	Ns	523		
	Stage 1–2	**5**	**1.14–25**	**0.019**	Unfavorable OS	83		
	Stage 3–4	0.99	0.79–1.23	0.32	Ns	487		
	Grade 1–2	1.28	0.86–1.92	0.22	ns	203		
	Grade 3	0.95	0.74–1.22	0.71	ns	392		
	TP53 mutant	0.8	0.55–1.18	0.26	ns	124		
Prostate	Metastatic, unknown subtype	**n/a**	**n/a**	**< 0.001**	Unfavorable DFS	130	Nickerson, 2017	[83]

*Note:* Significant values (*p* < 0.05) are in bold.

Abbreviations: DFS, disease‐free survival; ER, estrogen receptor; HER2, human epithelial growth factor 2; LN, lymph node; ns, not significant; OS, overall survival; PR, progesterone receptor.

^a^ Survival metric not stated.

^b^ Data based on stained biopsies null for TET2 versus TET2 expressing.

Numerous other studies have also reported associations between low *TET2* transcript levels and poor survival [83, 121, 122], including OS in head and neck squamous cell carcinoma (HNSCC) (HR [95% CI] = 1.96 [1.01–3.77], *p* = 0.044) [121] and disease‐free survival in metastatic PCa (*p* < 0.001) [83] (Table 3). Nickerson and colleagues [83] suggested that *TET2* loss may play a direct role in PCa progression, reporting a discernible reduction in TET2 expression between normal prostate tissue (*n* = 29) and adjacent tumor tissue (*n* = 131), and a significant decrease in expression between primary tumors and metastatic tumors (*n* = 19) (*p* = 0.001). Likewise, Kamdar and colleagues [125] found that low *TET2* expression is associated with PCa metastasis and poor OS and demonstrated that the expression status of genes mediated by *TET2*‐mediated promoter methylation significantly associates with recurrence‐free survival in these patients (*n* = 423).

Diminution of TET2 and reduction in 5hmC also correlate with poor survival and disease progression in mouse models of both melanoma and lung cancer [80, 95]. Global genomic reduction in 5hmC, potentially mediated by loss of TET2 function, has been reported as an independent adverse prognostic marker for OS in a cohort of NSCLC patients (*n* = 208), with low 5hmC levels significantly associated with large tumor size and lymph node metastasis. However, Zhao et al. [124] report conflicting data in their NSCLC patient cohort (*n* = 108), where patients with a *TET2* mutation (*n* = 12) had a significantly longer OS compared to patients without *TET2* mutation (*n* = 96) (HR [95% CI] = 0.15 [0.03–0.9]) (Table 3). Low *TET2* expression is also significantly associated with longer OS in patients with intrahepatic cholangiocarcinoma (iCCA) (HR [95% CI] = 0.34 [0.15–0.75], *p* = 0.007) [123] (Table 3). With the exception of these iCCA and NSCLC cohorts, the prevailing evidence suggests that low TET2 transcript/protein expression is associated with a poor outcome across numerous solid human tumors.

## 5 Potential of Targeted Therapy for *TET2* ‐Mutant Disease

Zou et al. [126] demonstrated that the proliferation of *TET2*‐mutant leukemic cells can be largely reversed in mouse models through RNAi *MBD6* knockdown. MBD6 is a methyl‐CpG‐binding‐domain protein, which recognizes chromatin‐associated retrotransposon RNA 5mC [126]. MBD6 promoted a more open chromatin state and increased transcription in stem cells in malignancies with TET2 depletion, suggesting a potential therapeutic avenue for targeting *TET2*‐mutant hematological malignancies via the development of MBD6 inhibitors.

Furthermore, Stölzel et al. [3] identified an elderly patient with AML who presented with a 1.1 Mb *TET2* deletion in one allele, a *TET2* nonsense mutation in the other allele, and a *NPM1* insertion mutation, who exhibited an astonishing response to a single agent 5′‐Azacitidine (aza) originally given as palliation. Hypothesizing that cellular sensitivity to aza monotherapy could be affected by *TET2*‐mutant allele dosage, Stölzel et al. conducted a series of studies to further elucidate this theory.

Using the HEL AML cell line, which carries a monoallelic *TET2* deletion, Stölzel and colleagues generated a CRISPR knockout isogenic cell line pair with a biallelic *TET2* mutation. When treated in vitro with aza, biallelic TET2 knockout (*TET2* ^−/−^) clones had a significantly lower cloning efficiency and proliferation in liquid media than parental cell clones with a monoallelic *TET2* mutation (*TET2* ^+/−^). This phenomenon was not seen when cells were treated with daunorubicin or Ara‐C. Furthermore, in an orthotopic xenograft AML mouse model, *TET2* ^−/−^ AML cells were subjected to aza‐induced negative selection when co‐injected with *TET2* ^+/−^ AML cells, further implicating aza as an effective monotherapy for *TET2* null AML.

Stölzel et al. then analyzed data from patients enrolled in the para el Estudio de la Terapeútica en Hematologías Malignas (PETHEMA) FLUGAZA phase III clinical trial [127] to investigate whether biallelic *TET2* mutation affected patient outcome in response to aza treatment. In this trial, a total of 50 patients had *TET2* mutation, including 6 patients with biallelic mutation (VAF > 85%). Three AML patients with biallelic *TET2* mutation were randomized to the low‐dose Ara‐C plus fludarabine (FLUGA) arm, but none of these entered remission, and all had relatively short OS (17, 45, and 111 days) compared to those with monoallelic *TET2* mutation. Of those patients randomized to the aza arm, three were identified with biallelic *TET2* mutation. Of these, two achieved complete remission and had prolonged OS (579 and 767 days) compared to aza‐treated individuals with monoallelic *TET2* mutation.

Findings by Stölzel and colleagues suggest that biallelic *TET2* mutations confer hypersensitivity to aza as a single agent in an AML setting when compared to monoallelic *TET2* mutations. Investigating *TET2*‐mutant allele dosage and subsequent TET2 protein expression at diagnosis could therefore indicate a possible treatment strategy for AML patients.

In summary, it is clear that promising avenues exist regarding targeted therapies for *TET2*‐mutant and TET2‐depleted hematological malignancies, although there is a paucity of data considering targeted therapy for *TET2*‐mutant solid tumors.

## 6 Conclusions and Future Perspectives

There are substantial data supporting an etiological role for TET2 dysfunction in the development of hematopoietic malignancies, such as MDS, AML, and several lymphoma subtypes [40, 41, 68, 70, 72, 73, 74, 76]. *TET2* mutations are primarily initiating events in the development of these diseases, requiring additional genetic mutations for malignant transformation. Alterations in *TET2* are also reported in numerous solid tumors [10, 81, 82, 83, 90, 91, 93, 94, 96, 97, 99, 103, 104, 106], although how these mutations drive malignant transformation in these settings is not as broadly studied. Understanding of the role of *TET2* in solid tumors differs among researchers, perhaps in part due to the still limited nature of our current knowledge of the subject. For example, in PCa [83] and certain female‐associated cancers [82], *TET2* mutation associates with late‐stage disease and disease progression, rather than functioning as an initiating event as seen in myeloid and lymphoid disorders. *TET2* mutations not only have a functional role in cancer development, but also in other human conditions such as atherosclerosis and cardiovascular disease [34, 88]. Moreover, the use of transcript or protein levels as surrogates for *TET2* genetic mutation can lead to misleading or apparently discrepant results, highlighting the importance of comparing equivalent datasets.

Individuals with CHIP are predisposed to developing hematological malignancy, with up to 1% of these patients transforming per year [37]. Mutations in genes associated with CHIP are also present at varying degrees in patients diagnosed with solid tumors [85]. Coombs and colleagues [85] determined that mutations in these genes were significantly associated with increasing patient age (*p* < 0.001), implicating CHIP as an age‐associated condition in a solid tumor setting, as well as in hematopoietic disorders. Attenuation of TET2 also has a clear association with prognosis in many hematological malignancies and solid tumors and is associated with an unfavorable outcome in the majority of settings (Table 3). It is evident that *TET2*‐mutant allele dosage is an important factor to consider; however, further research is warranted to deduce the impact of *TET2*‐mutant allele dosage on survival in a pan‐cancer context.

Taken together, the prevailing evidence supports a role for mutant *TET2* in the pathogenesis and prognostication of numerous solid human cancers.

## Author Contributions

**Zoë L. Hawking:** conceptualization (equal), visualization (equal), writing – original draft (lead). **James M. Allan:** conceptualization (equal), data curation (supporting), formal analysis (supporting), funding acquisition (lead), project administration (lead), supervision (lead), visualization (equal), writing – original draft (supporting), writing – review and editing (equal).

## Conflicts of Interest

The authors declare no conflicts of interest.

## Data Availability

The authors have nothing to report.
